# Large-scale genotyping and phenotyping of a worldwide winter wheat genebank for its use in pre-breeding

**DOI:** 10.1038/s41597-022-01891-5

**Published:** 2022-12-26

**Authors:** Albert W. Schulthess, Sandip M. Kale, Yusheng Zhao, Abhishek Gogna, Maximilian Rembe, Norman Philipp, Fang Liu, Ulrike Beukert, Albrecht Serfling, Axel Himmelbach, Markus Oppermann, Stephan Weise, Philipp H. G. Boeven, Johannes Schacht, C. Friedrich H. Longin, Sonja Kollers, Nina Pfeiffer, Viktor Korzun, Anne Fiebig, Danuta Schüler, Matthias Lange, Uwe Scholz, Nils Stein, Martin Mascher, Jochen C. Reif

**Affiliations:** 1 Leibniz Institute of Plant Genetics and Crop Plant Research (IPK) Gatersleben, Seeland, Germany; 2 Julius Kühn Institute (Federal Research Centre for Cultivated Plants), Quedlinburg, Germany; 3 Limagrain GmbH, Peine-Rosenthal, Edemissen, Germany; 4 State Plant Breeding Institute, University of Hohenheim, Stuttgart, Germany; 5 KWS SAAT SE & Co. KGaA, Einbeck, Germany; 6 KWS LOCHOW GmbH, Bergen, Germany; 7 Center for Integrated Breeding Research (CiBreed), Georg-August-University, Göttingen, Germany; 8 German Centre for Integrative Biodiversity Research (iDiv) Halle-Jena-Leipzig, Leipzig, Germany; 9 Present Address: Carlsberg Research Laboratory, Copenhagen, Denmark; 10 Present Address: Key Laboratory of Plant Germplasm Enhancement and Specialty Agriculture, Wuhan Botanical Garden, Chinese Academy of Sciences, Wuhan, China

**Keywords:** Plant breeding, Phylogenomics, Agricultural genetics

## Abstract

Plant genetic resources (PGR) stored at genebanks are humanity’s crop diversity savings for the future. Information on PGR contrasted with modern cultivars is key to select PGR parents for pre-breeding. Genotyping-by-sequencing was performed for 7,745 winter wheat PGR samples from the German Federal *ex situ* genebank at IPK Gatersleben and for 325 modern cultivars. Whole-genome shotgun sequencing was carried out for 446 diverse PGR samples and 322 modern cultivars and lines. In 19 field trials, 7,683 PGR and 232 elite cultivars were characterized for resistance to yellow rust - one of the major threats to wheat worldwide. Yield breeding values of 707 PGR were estimated using hybrid crosses with 36 cultivars - an approach that reduces the lack of agronomic adaptation of PGR and provides better estimates of their contribution to yield breeding. Cross-validations support the interoperability between genomic and phenotypic data. The here presented data are a stepping stone to unlock the functional variation of PGR for European pre-breeding and are the basis for future breeding and research activities.

## Abstract (summary)

**Table:** 

Measurement(s)	genome sequence • wheat stripe rust disease resistance • grain yield trait
Technology Type(s)	genotyping-by-sequencing • whole genome shotgun sequencing • Manual scoring in natural and artificially infected fields • Combine harvester
Factor Type(s)	genotype • location
Sample Characteristic - Organism	Triticum aestivum
Sample Characteristic - Environment	agricultural field
Sample Characteristic - Location	Germany

## Background & Summary

Common wheat (*Triticum aestivum* L.) is one of the ‘*big three*’ crops supplying most of the calories for the world population^1^. Urban expansion at expenses of agricultural areas^2–4^, climate change^4–6^, environmental pollution and agroecosystem degradation^3,4,6^ threaten future food security. Furthermore, wheat yield improvements showed a significant stagnation during the last decade^6^ which can be attributed to an eroded diversity in elite breeding^7–9^. Historically, we have learned the ‘hard way’ that a narrow genetic basis in cultivated plants increases the risk of crop pandemics^10–12^. For instance, *Puccinia striiformis* f. sp. *tritici*, - the causal agent of yellow rust (YR) - causes severe yield losses^13^ and its recent pandemics broke down several resistance genes (*Yr)* that are widely deployed in modern wheat cultivars^10,14–16^. Pandemic races ‘PstS7’ and ‘PstS8’, a.k.a. ‘Warrior’ and ‘Kranich’, respectively, originated in the near-Himalayan region^10,15^ and spread worldwide during the last decade^10,15,16^. During 2020, ‘Warrior’ and ‘Kranich’ lost importance in Europe, where ‘PstS10’ was the most prevalent race group^16^, thus illustrating how fast YR populations can evolve in the field. Paving the way towards a more sustainable agriculture, the management and diversification of resistant mechanisms^17^ will be the ‘main weapon’ to confront the increased risk of YR infection occurrence expected for Europe and other temperate regions in a climate change context^18^.

Decreased genetic diversity of cultivated wheat could be boosted by rescuing the abandoned or unexplored diversity treasure hidden in the ~800 K wheat plant genetic resources (PGR) stored at Genebanks worldwide^19,20^. In this respect, the Nagoya Protocol of the Convention of Biological Biodiversity was conceived to promote the use and equitable sharing of benefits from PGR for sustainable development (https://www.cbd.int/abs/about/). In addition, genebank genomics have already demonstrated how ‘molecular passports’ create value in genebank management by providing precise knowledge that goes beyond the boundaries of classical descriptors^20,21^. Nevertheless, due to the scarcity of agriculture-relevant information available for PGR, breeders often end up randomly choosing PGR as parents in crosses: an untargeted approach with low return of investment^22^. Furthermore, the value of PGR for agriculture and breeding is always relative to what can be found in modern cultivars. On the one hand, PGR characterizations should be put into this context by doing side-by-side comparisons between PGR and the modern gene pool^23,24^. On the other hand, PGR-*versus*-cultivar comparisons are biased for complex traits such as yield, where PGR are at disadvantage for their general lack of agronomic adaptation. This lack of adaptation is corrected by evaluating hybrid crosses between PGR and modern cultivars^14,25,26^ – an intermediate background that allows the estimation of the yield breeding value of PGR^14,27^.

Our study relies on the winter wheat collection hosted at the German Federal *ex situ* Genebank of Agricultural and Horticultural Crops in Gatersleben (IPK genebank). With ~ 27 K *Triticum* sp. PGR, the IPK collection is one of the largest among EU-27 countries^19^. Genotyping-by-sequencing (GBS) was carried out for 7,745 PGR samples of the IPK genebank and 325 modern cultivars. Whole-genome shotgun sequencing (WGS, 3-fold coverage) was performed for 446 diverse PGR samples, 191 modern cultivars and 131 advanced breeding lines. YR resistance was scored across 19 field experiments for 7,683 PGR and 232 elite cultivars. A total of 26 yield experiments allowed the evaluation of the contribution of 707 diverse PGR to yield improvement using ‘Elite × PGR’ bridging crosses. To the best of our knowledge, no large-scale datasets have been made publicly available so far that contain interoperable genomic and agriculture-relevant information on wheat PGR. Raw and processed data as well as phenotypic- and genomic-based approaches to prove data quality and interoperability are made available here following the FAIR principles of data publication^28^. In our main companion study^14^, genomic data was used to study crop genetic diversity as well as to detect duplicates, mislabeling of PGR, gaps between European genebank collections, selective sweeps and alien introgressions introduced by breeding. Mining YR and genomic data identified potential PGR donors of new sources of resistance not yet used in breeding. Yield breeding values guided early pre-breeding activities and allowed the obtention of PGR-derived lines with competitive yield levels in field experiments. We expect that these data incentive additional data science and breeding activities that can further valorize PGR.

## Methods

### Plant material

#### Overview

Across datasets, experiments and crosses, wheat genotypes trace back to 9,145 PGR from the IPK genebank, 340 European elite cultivars, and 131 German advanced breeding lines. Passport information respecting scientific name, growth habit, geographical origin, as well as acquisition (‘TRI’ PGR), release (‘B’ PGR/elite cultivars), and obtention (elite breeding lines) date of the studied material were collected in our companion publication^14^. In more detail, information for PGR of the ‘TRI’ collection was accessed through the Genebank Information System (GBIS)^29^ as extended MCPD-format. Passport data of PGR from the ‘Boris 96’ panel^30^, i.e. ‘B’ collection; as well as information for elite cultivars and German breeding lines were compiled from different publications, online databases and website sources^14^. Not only for the IPK genebank but for genebanks worldwide in general, passport data are dynamic and are prone to change over time due to, for instance, genebank curation activities^20,21^. To deal with different versions of identifiers in passport data, the IPK genebank uses unique digital object identifiers (DOIs) that are fixed and can trace back plant material to the formerly requested IPK accessions and their information.

Taxonomically, almost all PGR were either explicitly declared/classified as *Triticum aestivum* L. (67.2%) or in general as *Triticum* sp. (32.3%), while the very small proportion of remaining PGR have either not been classified yet (0.4%), corresponded to tetraploid wheat species (0.08%) or were wheat interspecific hybrids (0.02%). All European elite genotypes (cultivars plus breeding lines) were classified as *Triticum aestivum* L.. Growth habits of databases were contrasted with own field observations and correspondingly updated in our companion work^14^. PGR are almost entirely composed of strictly winter types (98.8%) with a small proportion of facultative types (1.2%). Similarly, most European elite genotypes are of winter type (96.8%) plus some facultative (2.1%) and spring (1.1%) type wheats.

**Fig. 1:**
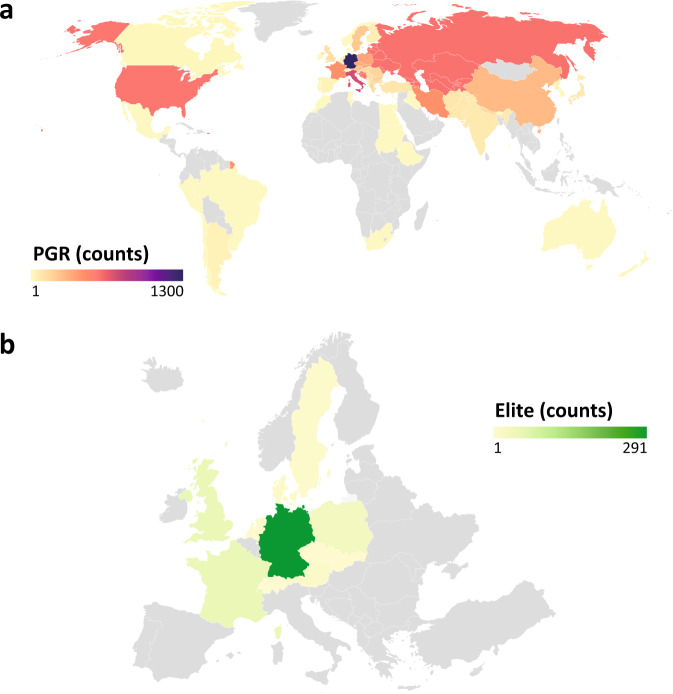
Number of counts according to the geographical origins of the 9,616 genotypes considered in the current study. (**a**) World map of 7,639 out of 9,145 winter wheat plant genetic resources (PGR) from the IPK genebank with 55 known geographical origins. (**b**) Map of Europe (excluding Russia), portraying releasing/obtention countries for 471 elite genotypes (340 European cultivars plus 131 German breeding lines). In (**a**) and (**b**), territories in gray lack entries. All maps were generated with Datawrapper.

Among the 55 different geographical origins reported for PGR (Fig. 1a), 52 have official ISO 3166 Alpha-3 country codes (https://www.iso.org/obp/ui). Current states/countries of the former Soviet Union (SUN), Yugoslavia (YUG), and Czechoslovakia (CSK) were pooled together according to each of these three origins to homogenize different nomenclatures that arise due to historical reasons. In addition, for 1,506 PGR (16.5%) their origins are unknown, while only one European cultivar (0.3%) is missing its exact country of origin. European nations were the most common PGR origins (60.6%), followed by Asia (15.1%), and American countries (7.4%) (Fig. 1a). Most European PGR originated in Germany (23.4% of European origins), Italy (13.3%), and the former Soviet Union (10.7%). The majority of European elite genotypes belongs to Germany (61.8%), Great Britain (11%), France (10.6%), and Poland (7%) (Fig. 1b).

**Fig. 2:**
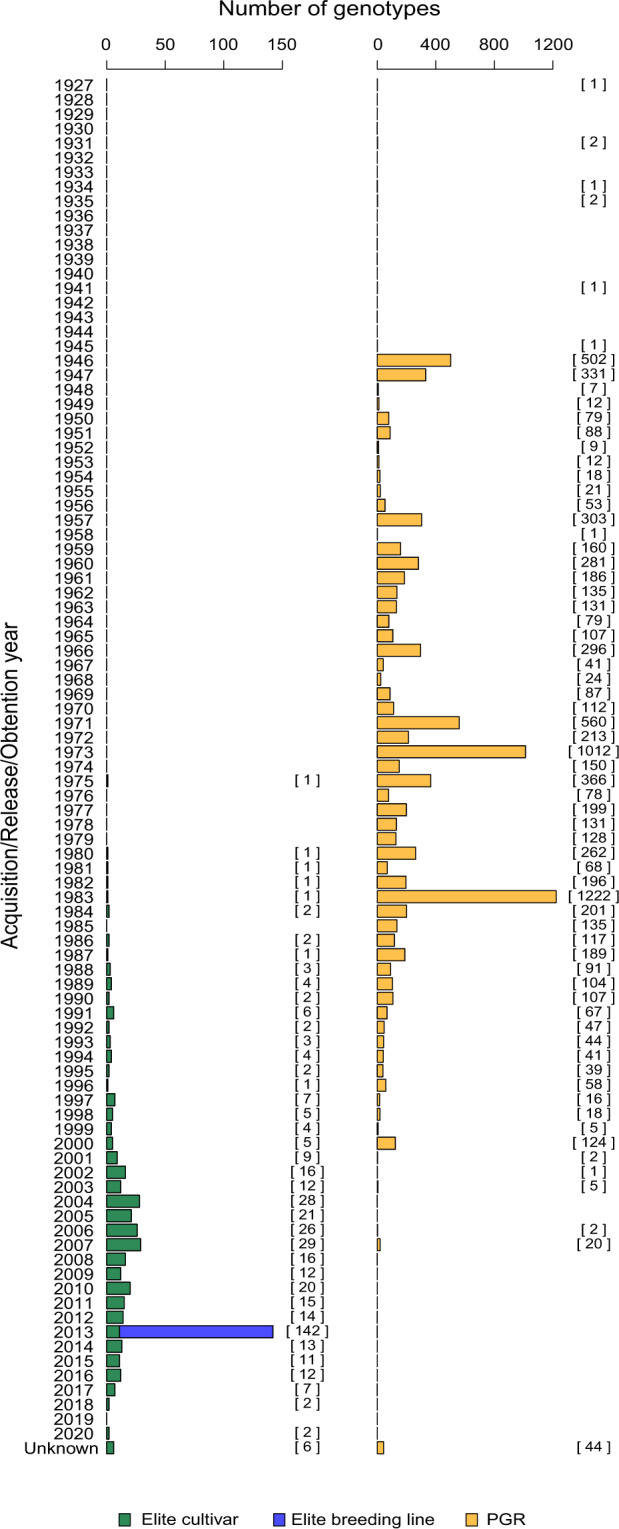
Distribution of the number of PGR (orange columns), elite cultivars (green) and breeding lines (blue) considered in this study according to their years of acquisition, release or obtention. When present, the exact counts number of genotypes per year are included within brackets [].

Regarding acquisition/release dates, these are unknown for 44 PGR (0.5%) and six elite cultivars (1.8%), respectively. PGR span an 80-year time window (1927–2007), with most of them (97.8%) tracing back to the last century (Fig. 2). Release or obtention dates of European elite genotypes range from 1975 to 2020, with the majority of them (87.5%) released/obtained from 2000 onwards. Among European cultivars, ‘Monopol’ is the oldest one (released in 1975) and is still grown today in Germany for its high milling and baking quality^31^, while ‘LGCharacter’ and ‘RGTRitter’ were the newest varieties (both released in 2020).

#### Seed sources

PGR from the ‘TRI’ catalog were directly obtained from the IPK genebank through GBIS, while seeds of PGR from the ‘B’ collection were kindly facilitated by Dr. Andreas Börner. The IPK genebank can normally provide ~5 g of seeds per PGR. Thus, in order to fulfill seed amount needs of large-scale research activities, seeds of 9,135 PGR were multiplied in a first step using single-row propagation plots. In parallel, 173 of these PGR plus 10 additional ones were multiplied under greenhouse conditions under the frame of the GenDiv project^32^. For elite cultivars, seeds were obtained from the local market either recently or in previous projects^33,34^. Seeds of the advanced breeding lines were provided by four breeding companies with base in Germany^35,36^.

For field-propagated PGR, one (two) representative ear(s) was (were) bagged in case of homogeneous (clearly non-homogenous) PGR. Following a single-seed descent (SSD) method, seeds from isolated ears were harvested separately from the rest of the plot and further propagated using an ear-to-row method. Hereafter, we refer to these PGR samples as SSD-PGR. Greenhouse-propagated PGR were fixed by applying the SSD method for two consecutive generations (2 × SSD) for each PGR^32^.

### Large-scale genotyping

#### Genomic data production

GBS was performed for 7,745 SSD-PGR plus 325 elite cultivars, while WGS was conducted for 263 SSD-PGR, 191 elite cultivars, 131 advanced breeding lines, and the 183 greenhouse-propagated PGR. GBS-characterized SSD-PGR trace back to 7,651 accessions and are identified with a unique correlative number combined with the PGR name nomenclature from the ‘TRI’ and ‘B’ collections. In more detail, SSD-PGR originate from single representative plants of 7,557 homogenous PGR and 94 double sampled non-homogenous PGR. For 171 modern cultivars, three plants per genotype were sequenced in a previous work^7^, while only a single plant per genotype was sequenced for the remaining samples. Regarding WGS-characterized SSD-PGR, only two of them trace back to a double sampled non-homogenous PGR. For each genotype, DNA was extracted from a single in-greenhouse-grown-10-days-old seedling using silica-membrane technology (NucleoSpin^®^ 96 Plant II) according to manufacturer instructions (Machery-Nagel).

GBS was conducted following a two-enzyme digestion protocol^37,38^. For this, DNA samples were simultaneously digested with PstI and MspI (New England Biolabs) and sticky ends were ligated with adapters containing sample-specific barcode sequences. This step allowed to track down each processed barcoded sample after DNA pooling. DNA was pooled into batches of 540 genotypes in equimolar amount to form a GBS library. Single-end sequencing (1 × 107 cycles) was conducted following Illumina protocols on HiSeq 2000 (171 × 3 = 513 samples), HiSeq 2500 (6,262 samples) or NovaSeq 6000 (1,637 samples) devices and using custom sequencing primers.

For WGS, libraries were prepared using the Nextera DNA Flex Library Prep following Illumina protocols and pooled afterwards in an equimolar manner. The multiplexed pool was quantified by qPCR and paired-end sequenced (2 × 151 cycles and 10 bp for the index reads) using a NovaSeq 6000 system and an average genome coverage of 3-fold.

#### Genomic data processing

In a first step, reads of each of the 171 modern cultivars sequenced in triplicate were pooled according to each original genotype. In this regard and if the opposite is not stated, the terms samples and genotypes are used indistinctly when referring to genomic data throughout the whole manuscript. Low-quality bases and adapter sequences were discarded from GBS raw reads using cutadapt (v1.16)^39^ by considering a minimum read length of 30 bp. This step was subsequently confirmed by using FastQC (v0.11.7)^40^. After this, BWA-MEM (v0.7.17)^41^ was applied at default settings to align the retained high-quality reads against the hexaploid wheat reference genome assembly of Chinese Spring (RefSeq v1.0)^42^. For WGS, reads were aligned with minimap2 (v2.17)^43^, in which the genome index size was set to 50 Gb while all other parameters remained as per default. Alignments were converted into binary map format using SAMtools (v1.9)^44^ and sorted afterwards with NovoSort^®^ (v3.06.05). Variant calling was done using the mpileup and call functions from SAMtools/BCFtools (v1.8)^45^ with parameters -DV and minimum read quality (q) cutoff = 20. Later, those biallelic variants were retained in the generated *variant calling format* (VCF) files using a custom awk script if the minimum QUAL = 40, minimum read depth for homozygous calls = 2 and for heterozygous calls = 4, in case of GBS, while these parameters were set to 40, 1, and 2, respectively, for WGS. From here onwards, we refer to these VCF files for GBS and WGS polymorphic variants as “unfiltered” SNP data. For the assessment of molecular diversity, linkage disequilibrium and genomic-phenotypic data interoperability (see last two sections of Methods), VCF files were further filtered using BCFtools and base and data.table (v1.12.8) functions in R environment^46^ (v3.6.1). Files were imported into R using the vcfR package (v1.12.0)^47^. Here, only markers having a minimum QUAL value of 40, a maximum percentage of missing values of 10%, ≥10 genotypes carrying any of both alleles in homozygous state, and ≤1% heterozygosity, were retained.

### Field evaluations of yellow rust resistance

Two groups of field experiments, summing up to 19 in total, were conducted to evaluate the resistance against YR (Table 1):

**Table 1:** Summary of the 12 unbalanced large-scale screening experiments and seven balanced precision experiments conducted in the field to evaluate yellow rust (YR, *Puccinia striiformis* f. sp. tritici) severity of plant genetic resources and elite cultivars.

Group	Experiment	Location^a^	Year^b^	Times^c^	Infection^d^	Agronomy^e^	Design^f^	Plot size (m^2^)	Replicates^g^	Entries^h^
**Large-scale screening**	**GAT_2014_1**	Gatersleben	2014–2015	1x	Natural	H	α lattice	0.4	2	1537
	**SST_2014_1**	Schackstedt	2014–2015	1x	Natural	H	α lattice	0.4	2	1537
	**GAT_2014_2**	Gatersleben	2014–2015	1x	Natural	H	α lattice	0.4	2	1560
	**GAT_2015_2**	Gatersleben	2015–2016	1x	Natural	H	α lattice	0.4	2	1561
	**SST_2015_2**	Schackstedt	2015–2016	1x	Natural	H	α lattice	0.4	2	1509
	**GAT_2015_3**	Gatersleben	2015–2016	1x	Natural	H	α lattice	0.4	2	1600
	**GAT_2016_3**	Gatersleben	2016–2017	1x	Natural	H	α lattice	0.4	2	1588
	**GAT_2017_3**	Gatersleben	2017–2018	1x	Natural	H	α lattice	0.4	2	1583
	**GAT_2017_5**	Gatersleben	2017–2018	1x	Natural	H	α lattice	0.4	2	1447
	**SST_2018_5**	Schackstedt	2018–2019	1x	Natural	H	α lattice	0.4	2	1428
	**GAT_2019_6**	Gatersleben	2019–2020	M:2x	Natural	H	α lattice	0.4	2	1697
	**SST_2019_6**	Schackstedt	2019–2020	1x	Natural	H	α lattice	0.4	1^(2)^	1696
**Precision**	**GAT_YR_2018**	Gatersleben	2018–2019	2x	Natural	H	α lattice	0.4	2	793
	**ROS_YR_2018**	Rosenthal	2018–2019	1x	Artificial „F“	H/F	α lattice	0.3	2	800
	**WTZ_YR_2018**	Wetze	2018–2019	2x	Artificial „S“	H/F	α lattice	0.5	2	800
	**GAT_YR_2019**	Gatersleben	2019–2020	2x	Natural	H	α lattice	0.4	2	801
	**QLB_YR_2019**	Quedlinburg	2019–2020	2x	Artificial „S“	F	IB	0.2	1	800
	**ROS_YR_2019**	Rosenthal	2019–2020	1x	Artificial „F“	H/F	α lattice	0.3	2	798
	**WTZ_YR_2019**	Wetze	2019–2020	1x	Artificial „S“	H/F	α lattice	0.5	2	799

^a^Location specificities are as follows: **Gatersleben** (latitude 51° 49’ 19.74” N, longitude 11° 17’ 11.80” E, 111 m.a.s.l., black soil of clayey loam-texture, 9 °C average annual temperature, 490 mm average annual rainfall), **Schackstedt** (latitude 51° 43’ 0” N, longitude 11° 37’ 0” E, 122 m.a.s.l., black soil of clayey loam-texture, 8.9 °C average annual temperature, 483 mm average annual rainfall), **Quedlinburg** (latitude 51° 46’ 22.22” N, longitude 11° 9’ 12.82” E, 140 m.a.s.l., black soil of clayey loam-texture, 8.9 °C average annual temperature, 497 mm average annual rainfall), **Rosenthal** (latitude 52° 18’ 10.242” N, longitude 10° 10’ 26.2668” E, 72 m.a.s.l., brown soil of slightly sandy loam-texture, 9.5 °C average annual temperature, 637 mm average annual rainfall), **Wetze** (latitude 51° 44’ 22.686” N, longitude 9° 54’ 36.1224” E, 136 m.a.s.l., brown soil of slightly clayey loam-texture, 8.6 °C average annual temperature, 664 mm average annual rainfall).

^b^Sowing - harvest years.

^c^Disease symptoms were scored either once (1x) or twice (2x) after the onset of YR infection. M:2x means that only the maximum infection value was recorded.

^d^Material was tested based either on natural infections (Natural), artificial inoculations using spreader plots (Artifial “S”) or full experiment artificial inoculations (Artifial “F”).

^e^Crop management considered chemical control against weeds (H) and/or use of fertilizers (F).

^f^Spatial variation was corrected using an alpha (α lattice) design with blocks subdivided into incomplete blocks or only considering incomplete blocks (IB).

^g^In case of 1^(2)^ only one of two replicates was measured.

^h^Number of entries according to the original field plan.

#### Large-scale resistance screening in unbalanced experiments

Twelve experiments were performed to large-scale evaluate the YR resistance of 7,684 PGR and 80 European elite cultivars based on naturally occurring infections (Table 1). An additional wheat genotype denoted as ‘Filler’ was considered due to technical reasons during sowing, but it was not part of the tested entries and has no passport data. Given the large number of entries to be screened, the plant material was tested in an unbalanced fashion by considering 1,428–1,697 entries per experiment (Table 1). Experiments were conducted between harvest years 2015–2020 at locations Gatersleben and Schackstedt. Wheat plants were cultivated in all experiments under rainfed continental conditions predominant at both German locations. In all experiments, chemical crop protection comprised only the use of herbicides, while no fertilizers were applied. In each experiment, the experimental unit corresponded to a 0.4 m^2^ plot. An alpha lattice design with two complete replications divided into incomplete blocks was used to account for uncontrolled spatial variation. Except experiment SST_2019_6, in which infection severity was scored in only one replication, both replications were measured in each experiment. In addition, GAT_2019_6 is the only experiment of this group in which YR infections were scored at two (early and late) dates. For this experiment, only the maximum as the most differentiating value among the two dates was retained for each plot. Otherwise experiments considered only a single scoring date after the clear onset of YR infections. Infection severity was expressed in a 1 (no symptoms) to 9 (severe infection) scoring scale following the official protocols of the German Federal Plant Variety Office^48^.

#### Precision balanced experiments

Seven experiments were conducted to test 200 European elite cultivars and 600 SSD-PGR (Table 1). Elite cultivars were pre-selected based on their maximized genomic diversity. The 600 SSD-PGR are not only a highly diverse sample but harbor also an increased proportion of resistant genotypes - which are in general at low frequencies in genebanks^14^. Among the 600 SSD-PGR, only two of them trace back to a double sampled non-homogenous PGR. Three wheat entries not belonging to the 200 cultivars plus 600 SSD-PGR but used to estimate experiment effects lack of passport data and were thus denoted as Filler_1–3. Experiments were conducted during harvest years 2019 and 2020 in German locations Gatersleben, Quedlinburg, Wetze, and Rosenthal (Table 1). Wheat plants were cultivated in all experiments under rainfed continental conditions predominant at all considered locations. Experiments GAT_YR_2018 and -_2019 were based on natural infections, while the other five experiments were artificially inoculated. Experiments ROS_YR_2018 and -_2019 relied on inoculations directly applied on the tested material, whereas surrounding susceptible spreader plots served as initial inoculum source for the tested entries in the other three inoculated experiments. Artificial inoculations were based on spore mixtures of race isolates from genetic groups ‘PstS7’ and ‘PstS10’ collected during past crop seasons. As reported by the Global Rust Reference Center^16^, these two aggressive race groups are virulent against resistance genes *Yr1*, *Yr2*, *Yr3*, *Yr4*, *Yr6*, *Yr7*, *Yr9*, *Yr17*, *Yr25*, *Yr32* and also against resistance specificities of genotypes ‘Spalding Prolific’ and ‘Avocet S’. As well, both race groups are avirulent against resistance genes *Yr5*, *Yr8*, *Yr10*, *Yr15*, *Yr24* and *Yr27*. In particular, ‘PstS7’ is virulent against the resistance specificity of the genotype ‘Ambition’, while ‘PstS10’ being avirulent. Experiments conducted in Gatersleben considered chemical weed control without use of fertilizers, while fertilizers but no herbicides were applied in the QLB_YR_2019 experiment (Table 1). Regarding experiments conducted in Wetze and Rosenthal both, herbicides and fertilizers were applied. Except for the QLB_YR_2019 experiment, where plant material was tested using a single replication in incomplete blocks, all experiments considered two complete replications and an alpha lattice design. The size of the experimental unit was a 0.2–0.5 m^2^ plot, with a size fixed for each test location. Disease symptoms were scored at a single timepoint after the onset of YR infection in WTZ_YR_2019, ROS_YR_2018 and -_2019 experiments, while early and late infections were recorded in the other four experiments. QLB_YR_2019 was the only experiment where infection was originally recorded using a percentage instead of a 1–9 scoring scale. Percentage scorings were transformed into a 1–9 scale using the scale conversion key of the German Federal Plant Variety Office^48^.

### Yield evaluations for informed pre-breeding

Two groups of field experiments, summing up to 26 in total, were conducted to evaluate the contribution of PGR to yield improvement using ‘Elite × PGR’ crosses (Table 2):

**Table 2:** Summary of experimental setup for the 22 and four estimation and validation experiments, respectively, of yield breeding values of plant genetic resources using bridging ‘Elite × PGR’ crosses.

Group	Series	Experiment	Location^a^	Year^b^	Trials^c^	Trial Design	Test^d^	Block size^e^	Entries^f^
**Estimation**	S1	**BOH_2015**	Böhnhausen	2015–2016	2	α lattice	U	9	611
	S1	**GAT_2015**	Gatersleben	2015–2016	2	α lattice	U	9	611
	S1	**HOH_2015**	Hohenheim	2015–2016	2	α lattice	U	9	611
	S1	**RNG_2015**	Renningen	2015–2016	2	α lattice	U	9	611
	S1	**SST_2015**	Schackstedt	2015–2016	2	α lattice	U	9	611
	S2	**ASD_2016**	Asendorf	2016–2017	2	α lattice	U	10	615
	S2	**GAT_2016**	Gatersleben	2016–2017	2	α lattice	U	10	614
	S2	**HOH_2016**	Hohenheim	2016–2017	2	α lattice	U	10	614
	S2	**RNG_2016**	Renningen	2016–2017	2	α lattice	U	10	617
	S2	**SST_2016**	Schackstedt	2016–2017	2	α lattice	U	10	617
	S3	**GAT_2017**	Gatersleben	2017–2018	1	α lattice	PR	10	433
	S3	**HDM_2017**	Hadmersleben	2017–2018	1	α lattice	U	10	500
	S3	**HOH_2017**	Hohenheim	2017–2018	1	α lattice	U	10	500
	S3	**RNG_2017**	Renningen	2017–2018	1	α lattice	U	10	500
	S3	**SST_2017**	Schackstedt	2017–2018	1	α lattice	PR	10	389
	S4	**GAT_2018**	Gatersleben	2018–2019	1	α lattice	U	12	495
	S4	**HDM_2018**	Hadmersleben	2018–2019	1	α lattice	U	12	488
	S4	**HOH_2018**	Hohenheim	2018–2019	1	α lattice	U	12	502
	S4	**RNG_2018**	Renningen	2018–2019	1	α lattice	U	12	502
	S4	**SST_2018**	Schackstedt	2018–2019	1	α lattice	U	12	502
	S5	**GAT_2019**	Gatersleben	2019–2020	1	α lattice	CR	10	510
	S5	**SST_2019**	Schackstedt	2019–2020	1	α lattice	CR	10	510
**Validation**	—	**GAT_PB_2019**	Gatersleben	2019–2020	1	Incomplete block	PR	4–5	95
	—	**SST_PB_2019**	Schackstedt	2019–2020	1	Incomplete block	PR	4–5	59
	—	**GAT_PB_2020**	Gatersleben	2020–2021	1	Incomplete block	PR	5	118
	—	**SST_PB_2020**	Schackstedt	2020–2021	1	Incomplete block	PR	5	108

^a^Locations specificities are as follows: **Böhnhausen** (latitude 51° 51’ 30.95” N, longitude 10° 57’ 44.32” E, 173 m.a.s.l.), **Gatersleben** (latitude 51° 49’ 19.74” N, longitude 11° 17’ 11.80” E, 111 m.a.s.l.), **Hohenheim** (latitude 49° 43’ 2.65” N, longitude 9° 11’ 12.70” E, 406 m.a.s.l.), **Renningen** (latitude 48° 44’ 29.58” N, longitude 8° 55’ 15.35” E, 484 m.a.s.l.), **Schackstedt** (latitude 51° 43’ 0” N, longitude 11° 37’ 0” E, 122 m.a.s.l.), **Asendorf** (latitude 52° 44’ 17.93” N, longitude 9° 0’ 24.11” E, 45 m.a.s.l.), **Hadmersleben** (latitude 51° 59’ 29.79” N, longitude 11° 18’ 12.79” E, 91 m.a.s.l.).

^b^Sowing - harvest years.

^c^Number of trials per experiment.

^d^Genotypes were tested either in an unreplicated (U), partially (>20%) replicated (PR) or completely replicated (CR) fashion.

^e^Size of the incomplete blocks (number of plots) used to account and correct for uncontrolled spatial variation. Plot sizes ranged between 6 to 9 m^2^.

^f^Number of entries according to the original field plan.

#### Yield breeding value estimation experiments

A total of 751 PGR - 234 PGR plus 527 SSD-PGR denoted with the suffix “_ISO” - and four elite cultivars were crossed as male parents with up to 16 out of 42 elite cultivars using chemical hybridization agents in the field. Particularly, 1,569 out of the 1,577 resulting hybrids corresponded to ‘Elite × PGR’ factorial crosses, while the remaining eight hybrids were ‘Elite_1_ × Elite_2_’ crosses. PGR serving as pollen donors comprise a diverse sample from the IPK genebank^14^ and were pre-selected for their pronounced anther extrusion based on large-scale screenings of genebank material. This pre-selection ensured a sufficient quantity of field-produced hybrid seed to be able to conduct multiple field experiments. Hybrid seed of ‘Elite × PGR’ and ‘Elite_1_ × Elite_2_’ crosses was produced at the IPK facilities. Sterility of the female parents was checked by bagging at least one plant per crossing block^49^. In addition, during the season following seed production, the uniformity and hybridity – a clear morphological differentiation from the female parent – of F_1_ seeds were controlled by growing each hybrid and its both parents side-by-side in 0.2 m^2^ plots. In parallel, the 1,577 IPK hybrids were tested together with 347 hybrids from the State Plant Breeding Institute of the University of Hohenheim (Landessaatzuchtanstalt, LSA), 518 parent genotypes, in addition to a set of 40 checks for their grain yield. Yield testing was conducted in a staggered fashion throughout five consecutive years by using partially overlapping entry groups (series), each composed of 501 to 617 genotypes (Table 2). Except for series 5, which was tested in only two locations, each series was tested in five locations. Across series, a total of 22 estimation experiments spanned together harvest years 2016–2020 and seven different German locations: Hohenheim, Renningen, Gatersleben, Schackstedt, Böhnhausen, Asendorf, and Hadmersleben. All experiments were conducted following an alpha lattice design. Experiments were performed either in an unreplicated (series 1, 2 and 4, plus three experiments of series 3), partially replicated (series 3) or completely replicated (series 5) fashion. For experiments of series 1 and 2, trialing and blocking was used to account and correct for uncontrolled spatial variation, while complete and/or incomplete blocks were considered for this purpose in series 3 to 5. The experimental unit corresponded for all series to a plot, with sizes ranging between 6 to 9 m^2^. Wheat plants were cultivated in all experiments under rainfed continental conditions predominant at all considered locations. In all breeding value estimation experiments plots were treated with fertilizers, herbicides, and pesticides by following conventional local practices. Harvest of plots was performed with a combine harvester and plot yield was adjusted to a 140 g H_2_O kg^−1^ moisture basis and expressed in Mg ha^−1^.

#### Yield breeding value validation experiments

The feasibility to develop high yielding pre-breeding material using breeding values as a tool for PGR parent selection was evaluated in early yield testing experiments (Table 2). Preliminary breeding values obtained from estimation experiments of harvest year 2016 were used to select 13 PGR with high yield breeding value estimates. These PGR served as pollen donors in 14 and 18 initial crosses during 2016 and 2017, respectively. Two additional PGR lacking of breeding value estimates were also considered as male parents in crosses during 2017. A set of 12 locally adapted European elite cultivars released between years 2004 and 2015 were used as pollen receptors in two- (Elite_1_ × PGR) and three-way crosses (Elite_2_ × [Elite_1_ × PGR]) involving PGR. Seeds of segregating progenies from each of the eight and 27 performed two- and three-way crosses, respectively, were increased and genetically fixed by two generations of selfing in Gatersleben. Besides roughing of off-types plus fixing true types, two-stage selection based on visual assessment of single plants, followed by rows focusing on plant height and leaf health in 0.5 m^2^ plots, was applied. Other than herbicides, no additional chemical treatments (i.e. fungicides, nitrogen fertilizers, etc.) were used for crop management during plant material depuration. After these selection steps, at least one genotype per initial cross could enter early yield testing experiments, summing up to a total of 189 advanced F_3:4_ families across 35 initial crosses. In the breeding value validation experiments (Table 2), candidate families were evaluated for their yield *per se* performance together with 15 elite checks under conventional local agricultural practices. Experiments were conducted during harvest years 2020 and 2021; with each year considering two locations: Gatersleben and Schackstedt. Wheat plants were cultivated in all experiments under rainfed continental conditions predominant at both considered locations. Elite checks corresponded to winter wheat cultivars approved for commercial use in Germany, with the French cultivar ‘Arezzo’ (released in 2007) being the oldest one, while the German ‘LGCharacter’ and French ‘RGTRitter’ varieties (both released in 2020) were the newest ones. Check varieties ‘RGTReform’, ‘Benchmark’, and ‘LGInformer’, were commercially released in 2014, 2015, and 2018, respectively, and connected the four validation experiments, thus allowing an integrated analysis. Seven additional genotypes (coded as LSA_1–7) present in early yield experiments were lines from the LSA breeding program. Although LSA lines lack of passport data, these were kept in datasets to not disrupt the estimation of field design effects. In all experiments, the experimental unit corresponded to a 6 m^2^ plot. Entries were tested in a partially replicated fashion and an incomplete block design was used to correct for uncontrolled spatial variation. Plots were harvested using a combine harvester, whereas grain yield was adjusted to a 140 g H_2_O kg^−1^ moisture basis and expressed in Mg ha^−1^.

### Phenotypic data analyses

A multiple-step strategy based on mixed models^50^ was implemented for data curation and parameter estimation:

#### Data curation and preparation

With the exception of yield breeding value validation experiments as well as the YR evaluations of SST_2019_6 and QLB_YR_2019 experiments, phenotypic data were outlier-corrected first by using the following general mixed model:

1 $${\rm{Trait}} \sim \mu +{\rm{Genotypes}}+{\rm{Experiments}}+{\rm{Genotypes}}\times {\rm{Experiments}}+{\rm{Trials}}+{\rm{Replicates}}\left({\rm{Trials}}\right)+{\rm{Blocks}}\left({\rm{Replicates}}:{\rm{Trials}}\right)+{\rm{Error,}}$$

where the common mean (*μ*) and genotype effects were treated as fixed factors, whereas experiments and their multiplicative interactions with genotypes, trials nested within experiments, replicates nested within trials, incomplete blocks nested within replicates and trials, as well as the model error nested within experiments were assumed as random and normally distributed.

In case of YR evaluations, Eq. (1) was fitted experiment-wise for each scoring timepoint (single, early or late). Therefore, effects of trials, experiments, and their interactions with genotypes were dropped from Eq. (1) according to each specific experimental design (Table 1). Normalized residuals of this model were obtained by subtracting their average and dividing them by their standard deviation. After this, residuals were tested experiment-wise for their significance as outliers following Anscombe and Tukey^51^ and assuming a nominal alpha level of 0.01. Accordingly, datapoints flagged as outliers were discarded from final datasets.

For breeding value estimation experiments, outlier correction of yield data underwent series-wise and trials and/or replicate effects in Eq. (1) were considered/ignored according to the respective experimental design(s) used in each series (Table 2). Later, yield data were screened series-wise for significant outliers using the method M4 “Bonferroni-Holm with rescaled median absolute deviation standardized residuals” as suggested by Bernal-Vasquez *et al*.^52^. Following this, datapoints detected as significant outliers were accordingly discarded. Afterwards, yield records of series 1 to 4 were adjusted series-wise for trials, replicates and/or effects of incomplete blocks using Eq. (1) according to the experimental design(s) specific for each series (Table 2) while this adjustment underwent experiment-wise for series 5. In a next step, 161 hybrids (144 IPK plus 17 LSA hybrids) with low homogeneity and/or hybridity were discarded from the integrated dataset and IPK hybrids plus line parent genotypes were subtracted for parameter estimation.

#### Parameter estimations within experiments

Following data preparation, parameter estimation underwent first experiment-wise for YR-scores and yield breeding value validation experiments. Best linear unbiased estimations (BLUEs) of genotypes for YR-scores were computed for each scoring timepoint (“single”, “early” or “late”) of replicated experiments as well as for yield performance in each yield breeding value validation experiment. For this, effects of trials, experiments, and their interactions with genotypes were ignored in Eq. (1) and design effects were considered/skipped according to each specific experiment (Tables 1 and 2). Due to the absence of replications in QLB_YR_2019 and SST_2019_6, YR-scores in these particular experiments were adjusted out of the frame of mixed models using the means of corresponding incomplete blocks. In parallel, variance components of single replicated experiments were estimated for each scoring timepoint in a similar fashion as BLUEs but assuming genotypes as random. Variance estimates were used to compute experiment-specific heritabilities in the way:

2 $${h}_{{\rm{Experiment}}}^{2}=\frac{{\widehat{\sigma }}_{g}^{2}}{{\widehat{\sigma }}_{g}^{2}+\frac{{\widehat{\sigma }}_{Error}^{2}}{\overline{{\rm{N}}.{\rm{Rep}}.}}}$$

where ${\widehat{\sigma }}_{g}^{2}$ and ${\widehat{\sigma }}_{Error}^{2}$ are the genotypic and error variance estimates, respectively, while $\overline{{\rm{N}}.{\rm{Rep}}.}$ is the average number of effective replicates after considering missing plots and/or outlier-correction.

#### Parameter estimations across experiments

*Large-scale YR screening experiments*: firstly, a correlation test for BLUEs and/or experimental design corrected data was performed between experiments. Later, BLUEs of genotypes and variance components of YR-scores were obtained from the outlier-corrected data integrated across 12 experiments. For this, the trial effect was dropped from Eq. (1). The heritability across experiments was then computed as:

3 $${h}_{{\rm{Across}}}^{2}=\frac{{\widehat{\sigma }}_{g}^{2}}{{\widehat{\sigma }}_{g}^{2}+\frac{{\widehat{\sigma }}_{g\times Exp.}^{2}}{\overline{{\rm{N}}.{\rm{Exp}}.}}+\frac{{\widehat{\sigma }}_{\overline{Error}}^{2}}{\overline{{\rm{N}}.{\rm{Exp}}.}\times \overline{{\rm{N}}.{\rm{Rep}}.}}},$$

where ${\widehat{\sigma }}_{g\times Exp.}^{2}$ is the variance of the interaction between genotypes and experiments, ${\widehat{\sigma }}_{\overline{Error}}^{2}$ is the average error variance nested within experiments, $\overline{{\rm{N}}.{\rm{Exp}}.}$ is the average number of effective experiments used to test a genotype, while all other components in Eq. (3) retain the definitions from Eq. (2).

*Precision balanced YR experiments*: data of one genotype (‘PilgrimPZO’) was discarded from these integrated analyses due to material mislabeling. In a first step, correlations of BLUEs and/or experimental design corrected data were computed between experiments. In addition, the maximum value among early and late scorings or single timepoint scoring were selected experiment-wise for each genotype based on single experiment BLUEs or data corrected for incomplete-block effects in the case of QLB_YR_2019. Using this integrated dataset BLUEs were computed across experiments by fitting Eq. (1) but only considering *μ* as well as genotype, experiment and error effects. By assuming *μ* as fixed factor and the remaining model effects as random, ${\widehat{\sigma }}_{g}^{2}$ was obtained but the error term and genotype × experiment interaction were confounded in this model. Assuming that the average of single-experiment error variance estimates equals ${\widehat{\sigma }}_{\overline{Error}}^{2}$, ${\widehat{\sigma }}_{g\times Exp.}^{2}={\widehat{\sigma }}_{Error}^{2}-\frac{{\widehat{\sigma }}_{\overline{Error}}^{2}}{\overline{{\rm{N}}.{\rm{Rep}}.}}$, where ${\widehat{\sigma }}_{Error}^{2}$ is the variance estimate of the confounded error and interaction terms of the model. After this, Eq. (3) was used to estimate the heritability of YR-scores across precision experiments.

*Yield breeding value estimation experiments*: in a first step, correlations of BLUEs and/or experimental design corrected data were computed between experiments. Later, the following mixed model was fitted to the outlier-and-design corrected yield data from 22 estimation experiments:

4 $${\rm{Yield}} \sim {\rm{Type}}+{\rm{Experiments}}+{\rm{Lines}}+{\rm{Hybrids}}+{\rm{Lines}}\times {\rm{Experiments}}+{\rm{Hybrids}}\times {\rm{Experiments}}+{\rm{Error,}}$$

where Type includes the specific group mean of either lines (*μ*_Lines_) or hybrids (*μ*_Hybrids_) and was assumed as fixed, while hybrid and lines, experiments and their interactions with genotypes as well as the error nested within experiments were assumed as random factors. For lines, variance estimates ${\widehat{\sigma }}_{{g}_{Lines}}^{2}$ and ${\widehat{\sigma }}_{{g}_{Lines}\times Exp.}^{2}$ of yield *per se* performance are directly obtained from Eq. (4), while the total variance of hybrid yield can be further decomposed as follows:

5 $${{\rm{Yield}}}_{{\rm{Hybrids}}} \sim {\mu }_{{\rm{Hybrids}}}+{\rm{Experiments}}+{{\rm{GCA}}}_{{\rm{Females}}}+{{\rm{GCA}}}_{{\rm{Males}}}+{\rm{SCA}}+{{\rm{GCA}}}_{{\rm{Females}}}\times {\rm{Experiment}}+{{\rm{GCA}}}_{{\rm{Males}}}\times {\rm{Experiments}}+{\rm{Error,}}$$

where GCA_Females_ and GCA_Males_ are the general combining abilities (GCA) of female and male parents, respectively, SCA is the specific combining ability between parents, while the error term is confounded with the SCA×Experiments interaction. From Eq. (5), ${\widehat{\sigma }}_{{g}_{Hybrids}}^{2}={\widehat{\sigma }}_{GC{A}_{Females}}^{2}+{\widehat{\sigma }}_{GC{A}_{Males}}^{2}+{\widehat{\sigma }}_{SCA}^{2}$, and ${\widehat{\sigma }}_{{g}_{Hybrids}\times Exp.}^{2}={\widehat{\sigma }}_{GC{A}_{Females}\times Exp.}^{2}+{\widehat{\sigma }}_{GC{A}_{Males}\times Exp.}^{2}$, are derived. These estimates in addition to variance components of lines and ${\widehat{\sigma }}_{\overline{Error}}^{2}$, were used to compute yield heritabilities (${h}_{{\rm{Lines}}}^{2}$ and ${h}_{{\rm{Hybrids}}}^{2}$) across experiments according to Eq. (3). In parallel, the breeding value of the i^th^ male parent genotype was defined as ${\widehat{\mu }}_{{\rm{Hybrids}}}+{\overline{{\rm{GCA}}}}_{{\rm{Male}}({\rm{i}})}$, where ${\overline{{\rm{GCA}}}}_{{\rm{Male}}({\rm{i}})}$ is the best linear unbiased predictor (BLUP) of the corresponding male parent component. In case of PGR tested in hybrids as both PGR and SSD-PGR, the respective breeding values were averaged into a single estimate. The heritability of breeding values estimated in the hybrid context was defined as:

6 $${h}^{2}=\frac{{\widehat{\sigma }}_{GC{A}_{Males}}^{2}}{{\widehat{\sigma }}_{GC{A}_{Males}}^{2}+\frac{{\widehat{\sigma }}_{GC{A}_{Males}\times Exp.}^{2}}{\overline{{\rm{N}}.{\rm{Exp}}.}}+\frac{{\widehat{\sigma }}_{SCA}^{2}}{\overline{{\rm{N}}.{\rm{Fem}}.}}+\frac{{\widehat{\sigma }}_{\overline{Error}}^{2}}{\overline{{\rm{N}}.{\rm{Exp}}.}\times \overline{{\rm{N}}.{\rm{Rep}}.}}},$$

where $\overline{{\rm{N}}.{\rm{Fem}}}$. is the average number of crossing females used to test male parents, while the remaining terms retain all previous definitions.

*Yield breeding value validation experiments*: BLUEs of genotypes and variance components of the yield performance across breeding value validation experiments were obtained using Eq. (1) but skipping replicate and trial effects and assuming a common error variance for all experiments. The heritability of yield performance across experiments was then computed as specified in Eq. (3).

Linear mixed models of phenotypic data analyses were fitted using the average information matrix algorithm for restricted maximum likelihood (REML) computation implemented in ASReml-R (v3.0 and 4.0)^53,54^.

### Molecular diversity and linkage disequilibrium as captured by genotyping platforms

Considering the total number of genotypes for each genotyping platform (GBS and WGS) as *n* and the total number of filtered markers as *p*, SNP-matrices can be represented as *M* = (*m*_*si*_), with 1 ≤ *i* ≤ *n* and 1 ≤ *s* ≤ *p*. Given 1 ≤ *j* ≤ *n*, the Rogers’ distance^55^ between any *i*-th and *j*-th genotypes is calculated in the way: $\frac{1}{2p}{\sum }_{s=1}^{p}\left|{m}_{si}-{m}_{sj}\right|$. For each row of *M*, homozygous states for reference and alternative alleles were coded as 0 and 2 according to RefSeq v1.0, respectively, while 1 represented the heterozygous state. A principal coordinate analysis (PCoA) was conducted on Rogers’ distance matrices using the cmdscale() function in R. Here, the first two PCo, i.e. PCo1 and 2, were retained to respectively portray the molecular diversity captured by GBS and WGS using biplots. To investigate the level of concordance between GBS and WGS, a Mantel correlation test^56^ as implemented in the vegan R package (v2.5–7)^57^ was performed on the Rogers’ distance matrices for 454 overlapping genotypes between both platforms.

SNP filtering resulted in 29,846 GBS and 1,452,806 WGS markers having a minimum QUAL score of 40, a maximum missing value rate of 10%, ≥10 genotypes carrying any of both alleles in homozygous state, and up to 1% heterozygosity. This implied 24,091,446 and more than 67 billion intra-chromosomal marker combinations to be assessed for GBS and WGS platforms, respectively. To reduce the computational burden for WGS SNP markers, variants were chromosome-wise downsampled to an expected value of 10 markers per Mb, resulting in 145,307 markers across 21 chromosomes and the unanchored sequences. Intra-chromosomal linkage disequilibrium between marker (column) pairs of *t*(*M*) was estimated as the squared correlation coefficient (*r*^2^)^58^, while physical distances were computed as the pairwise Euclidean distance between SNP marker positions on RefSeq v1.0 of Chinese Spring^41^. Efficient correlation computation was performed using the bigcor() function implemented in the propagate R package (v1.0–6)^59^. After this, cubic splines were fitted in R environment using smooth.spline() to estimate the *r*^2^ decay as a function of the distances between marker pairs in different genetic pools: PGR samples, European elite cultivars and German elite breeding lines.

### Genomic-phenotypic data interoperability

The accuracy of the genomic best linear unbiased prediction (GBLUP)^60^ was used as a quality measure for data interoperability of overlapping phenotypic and genomic datasets. Using matrix nomenclature, the mixed model^50^ for GBLUP can be described as follows:

7 $${\bf{Y}}={{\bf{1}}}_{n}\mu +Z{\bf{g}}+{\bf{e}},$$

where **Y** denotes an *n*-dimensional vector of trait values for *n* genotypes, **1**_*n*_ is a unit vector of length *n*, *μ* indicates the fixed common population mean, Z corresponds to a design matrix connecting the elements of **g** to **Y**, **g** represents an *n*-dimensional vector of random genotypic values and **e** is the random residual term. Traits corresponded to either ready-to-use BLUEs of YR-scores across large-scale screening or precision experiments as well as ready-to-use yield breeding values of PGR estimated across experiments using ‘Elite × PGR’ F_1_ crosses. In Eq. (7), ${\bf{g}} \sim N({\bf{0}},{\sigma }_{g}^{2}G)$ and ${\bf{e}} \sim N\left({\bf{0}},{\sigma }_{e}^{2}I\right)$, where *G* is an additive genomic relationship matrix computed from markers based on the first method of VanRaden^60^, *I* indicates an identity matrix, while ${\sigma }_{g}^{2}$ and ${\sigma }_{e}^{2}$ correspond to the genotypic and error variance components of the model, respectively. For *G* matrix computation, profiles in *M* were coded as −1, 1, 0, for the reference and alternative alleles at homozygous and heterozygous states, respectively, while missing values were imputed using the average of observed values for each particular locus. Prediction accuracies of GBLUP were estimated by means of five-fold cross validations. For this, datasets containing both phenotypic and genomic data were randomly subdivided into five groups. The first four groups served together as the training set, whereas the fifth group corresponded to the prediction set. During prediction, the phenotypes of the prediction set were masked, while monomorphic markers across training and predictions sets were discarded for *G* matrix computation. After prediction, the accuracy was computed for genotypes in the prediction set as the Pearson correlation coefficient between predicted and observed values divided by $\sqrt{{h}^{2}}$, with *h*^2^ being the heritability of the corresponding predicted trait. The five groups were permuted, so that each of them serves exactly four times as training set, and one time as prediction set. The random subdivision into five groups was repeated 20 times, giving a total of 5 × 20 = 100 cross-validation runs.

Mixed model equations for genomic prediction were computed using REML as implemented in the rrBLUP R package (v4.6.1)^61^. All computational methods related to phenotypic analyses and genomic prediction were implemented within R statistical environment^46^ (v3.4.4, v3.6.1).

## Data Records

*Raw sequencing reads*: FASTQ files containing raw reads for 8,070 (GBS) and 768 (WGS) genotypes were deposited at the European Nucleotide Archive^62^: GBS^63^ and WGS^64,65^. Sequenced genotypes are findable through their ‘SAMEA’ IDs on EMBL-EBI BioSamples^66^: a full list of integrated GBS and WGS ‘SAMEA’ BioSample IDs connected with plant material passports, passport data sources, SSD- and IPK genebank DOIs was deposited at e!DAL-PGP^67^ and can be accessed here^68^.

*SNP markers*: variant calling results based on read mapping against the reference sequence of Chinese Spring (RefSeq v1.0)^42^ were stored as VCF files. Unfiltered VCF files for GBS (‘090222_8070_sample_unfiltered_snps_biosample.vcf.gz’) and WGS (‘070222_768_samples_wgs_no_filter_biosample.vcf.gz’) data are located at the European Nucleotide Archive and can be accessed here^69^. These files contain information on 1,628,276 and 213,804,916 SNP markers with a minimum QUAL = 40 and polymorphic among 8,070 GBS and 768 WGS samples, respectively. Genotype names are coded using the respective ‘SAMEA’ BioSample IDs^68^. VCF files contain relevant information for each SNP regarding its chromosome, physical position on chromosome in bp, reference and alternative alleles, as well as QUAL. R objects containing reduced numbers of SNP markers used for technical validations were deposited into the e!DAL-PGP repository and can be accessed here^70^.

Phenotypic records were also deposited into e!DAL-PGP:

*YR-scorings*: infection severity of *Puccinia striiformis* f. sp. *tritici* on wheat plants were field recorded on plots and expressed in a 1 (no symptoms) to 9 (severe infection) scoring scale following the official protocols of the German Federal Plant Variety Office^48^. Text files containing YR-scores were stored in ISA-Tab format. After outlier correction, the effective number of records from large-scale screening^71^ and precision experiments^72^ amount to 35,043 and 15,353, respectively. Besides phenotypic records, each ISA-Tab file includes information that connect records with the corresponding plant material, incomplete block, replication, year, location, and experiment, in addition to plant material passports, passport data sources, SSD- and IPK genebank DOIs, as well as ‘SAMEA’ IDs. Ready-to-use BLUEs computed across large-scale screening experiments^71^ for 7,682 PGR and 80 elite cultivars as well as BLUEs across precision experiments^72^ for 199 elite cultivars and 600 SSD-PGR are available in the’BLUEs_and_heritabilities‘ folder associated to each corresponding dataset.

*Yield records*: wheat grain yield was field recorded on plots and expressed in Mg ha^−1^ on a 140 g H_2_O kg^−1^ moisture basis. Text files containing grain yield records were stored in ISA-Tab format. After correction for outliers and design effects, the effective number of phenotypic records in breeding value estimation experiments amounts to 7,407^73^. The ISA-Tab file contains also information to connect phenotypic records with the corresponding tested material, material type (hybrid or line), parents, year, location, experiment, and series, in addition to plant material passports of parent lines, their passport data sources, SSD- and IPK genebank DOIs, as well as ‘SAMEA’ IDs. Ready-to-use breeding values computed across estimation experiments for 707 PGR can be found in the respective ‘Breeding_values_and_heritabilities’ folder^73^. For breeding value validation experiments, the number of yield records corresponds to 739^74^. The corresponding ISA-Tab file includes also the information needed to connect phenotypic records to the respective plant material, FAMILY-DOI, incomplete block, year, location, and experiment, in addition to plant material passports of check cultivars and parent lines, their passport data sources, SSD- and IPK genebank DOIs, as well as ‘SAMEA’ IDs. Ready-to-use BLUEs of grain yield computed across validation experiments are also available for 189 advanced F_3:4_ progenies and 15 elite cultivar checks in the corresponding ‘BLUEs_and_heritabilities’ folder^74^.

For more details on genomic and phenotypic data production, preparation, and processing, please refer to the Methods section. Machine readable details are also included in a ‘i_investigation.txt’ file associated to each phenotypic dataset^71–74^.

## Technical Validation

### Genotyping-by-sequencing is a precise and cost-efficient platform to study molecular diversity in genebanks

For GBS, the average total read count per genotype (after trimming) corresponded to ~2.64 million, while a WGS sample presented on average ~354.8 million reads (Table 3). These numbers slightly decreased to ~2.59 (98.3% of total) and ~349.1(98.4%) after read mapping against Chinese Spring (RefSeq v1.0)^42^, with 62.7% (GBS) and 47.7% (WGS) of the average read count per genotype having a mapping quality >q20. These reads (>q20) were retained during variant calling and allowed the obtention of VCF files for 8,070 GBS and 768 WGS samples, respectively. SNP markers with QUAL ≥40, ≤10% missing values rate, at least 10 genotypes carrying each allele in homozygous state, and a maximum of 1% heterozygosity, were used to assess the molecular diversity, linkage disequilibrium and genomic-phenotypic data interoperability. The latter can be found after quality assessment sections of phenotypic data. After filtering a total of 29,846 SNP markers across 8,070 genotypes were available for GBS, while the SNP-matrix for WGS contained 1,452,806 markers across 768 genotypes^70^.

**Table 3:** Minimum, maximum, median and average sequencing read counts per genotype characterized with genotyping-by-sequencing (GBS) or whole-genome sequencing (WGS, 3-fold coverage).

Reads	GBS (8,070 genotypes)			
	Minimum	Median	Average	Maximum
All^a^	778,809	2,406,622	2,637,070.2	27,663,505
Mapped				
Total	757,460	2,369,798.5	2,591,115.5	27,296,923
>q1	576,566	1,798,418.5	1,962,639.1	20,679,706
>q20	481,039	1,486,933	1,623,899	17,034,192
>q30	415,903	1,284,438.5	1,402,438	14,765,665
	**WGS (768 genotypes)**			
All	56,358,130	346,951,848	354,818,926.3	1,015,707,996
Mapped				
Total	55,439,721	341,737,943	349,082,726.1	999,714,679
>q10	27,616,390	178,233,289.5	178,555,659.1	473,928,110
>q20	25,815,724	166,330,159.5	166,505,569.7	439,321,203
>q30	24,084,200	154,655,202	154,777,065.9	406,186,889

Read counts are presented according to different minimum read mapping quality (q) levels.

^a^After trimming.

**Fig. 3:**
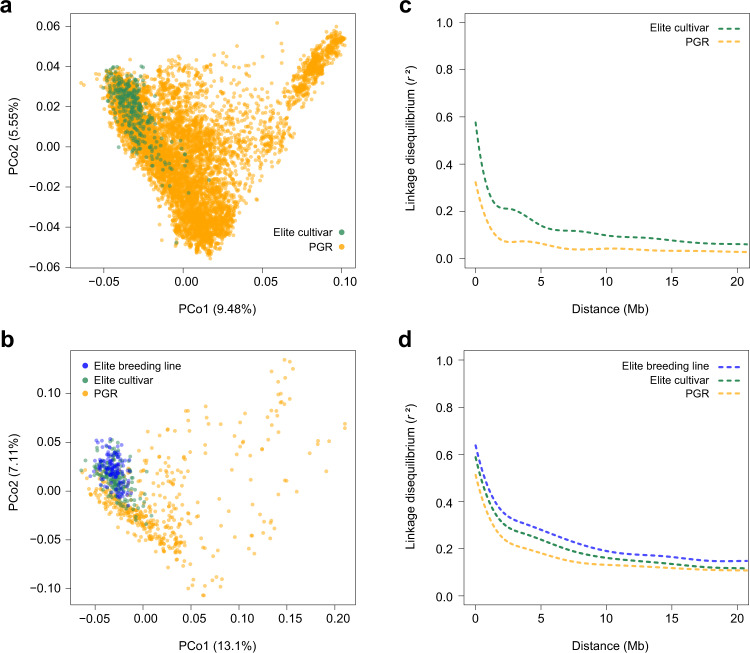
Molecular neutral diversity and linkage disequilibrium decay in genebank and elite plant material. Molecular diversity portrayed by the first two principal coordinates (PCos) from Rogers’ distance matrices calculated using genotyping-by-sequencing (GBS, (**a**)) and whole-genome sequencing (WGS, (**b**)). Intra-chromosomal linkage disequilibrium (*r*^2^) as a function of the genomic physical distance (Mb) in GBS (**c**) and WGS (**d**). GBS was conducted for 7,745 plant genetic resources (PGR) samples from the IPK genebank and 325 European elite cultivars. WGS was performed for 191 European elite cultivars, 131 German elite breeding lines and 446 PGR samples from the IPK genebank. Percentage of variation explained by PCos are included in brackets (). For *r*^2^ decay, distances between SNP pairs correspond to RefSeq v1.0 of Chinese Spring while cubic splines were fitted to whole genomes but only the first 20 Mb are portrayed.

A PCoA was conducted on the Rogers’ distances to assess the molecular diversity among the 8,070 GBS and the 768 WGS samples (Fig. 3a,b). Rogers’ distance matrices and a custom R code for their computation are also available here^70^. PCo1 and 2 explained 9.5% and 5.6% of the molecular variation portrayed by GBS-SNP markers, respectively, while the reduced number of samples for WGS slightly increased the percentage of explained variation to 13.1% (PCo1) and 7.1% (PCo2). As expected, PGR samples expand the molecular diversity of the elite pool, whose genotypes cluster very close to each other in the left corner of biplots. Coordinates of the German elite breeding lines formed a slightly more contracted group than elite cultivars (Fig. 3b). Nevertheless, the good overlap between these two latter groups reflects the continuous material exchange that takes place between European wheat breeders^75,76^.

The intra-chromosomal decay of linkage disequilibrium (*r*^2^) as a function of physical distance was estimated by fitting cubic splines curves for each genotypic group and genotyping platform (Fig. 3c,d). Independent of the genotyping platform and as already observed in past studies^77,78^, *r*^2^ values decay faster in genebank samples as compared to modern elite genotypes. European elite cultivars presented in turn a slightly faster decay of linkage disequilibrium compared to the German elite breeding lines (Fig. 3d). Since differences in *r*^2^ decay between GBS (Fig. 3c) and WGS (Fig. 3d) platforms were less pronounced for elite cultivars, we attribute the faster *r*^2^ decay in PGR samples portrayed by GBS to the large difference in population size (7,745 GBS vs 446 WGS samples) rather than to the genotyping platforms themselves.

As also reported in our companion study^14^, the correlation between GBS- and WGS-based Rogers’ distances among 454 genotypes characterized with both platforms amounted to 0.88 (Mantel correlation test p-value = 0.001). According to correlation estimates from past works comparing GBS with other mainstream genotyping platforms to assess crop plant genebank diversity^7,79^, the correspondence between GBS and WGS observed in our study is very high. This is noteworthy, considering that WGS reads deliver the least biased genome representation possible. GBS is a cost-effective and simple method that reduces the complexity of genomes. On the one hand, complexity reduction obviously limits the depth of analysis for large and complex genomes of species such as hexaploid wheat. On the other hand, if the primary objective of characterizations is the macro-assessment of molecular diversity and linkage-disequilibrium in wheat populations, GBS is the method of choice^7,79^.

### Large-scale screening and precision experiments revealed yellow rust resistance as a rare phenotype in the German Federal ex situ genebank

**Fig. 4:**
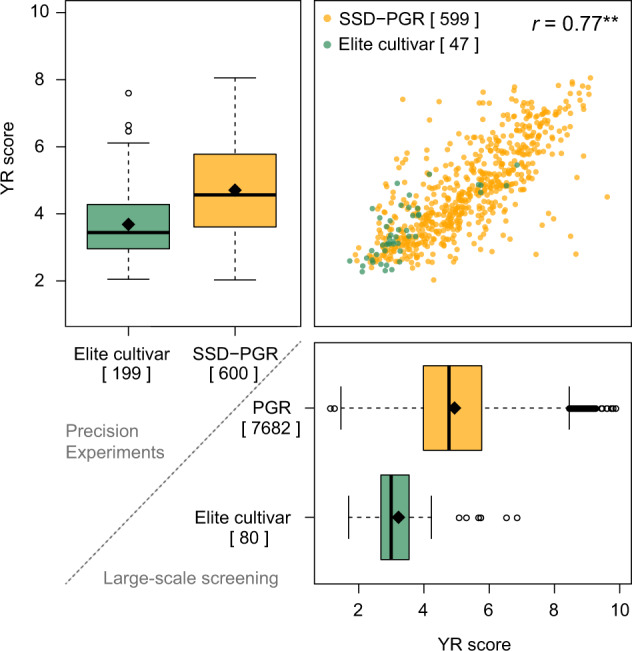
Distribution of the best linear unbiased estimations (BLUEs) across experiments for outlier-corrected yellow rust (YR, *Puccinia striiformis* f. sp. *tritici*) infections of plant genetic resources (PGR or SSD-PGR) and elite cultivars (Elite) tested in precision (boxplot, upper left corner), large-scale screening (boxplot, lower right) or both types of field experiments (scatter plot, upper right). YR infections were scored using an ordinal rating scale between 1 and 9, where 1 means complete absence of YR leaf symptoms and 9 denotes fully infected leaves. BLUEs that lie outside of the 1–9 parametric space are due to the unorthogonal structure of unbalanced experiments. In total, 19 field experiments were conducted between harvest years 2015 and 2020 considering five German locations. Large-scale screenings fully relied on natural YR infections, while five out of seven precision experiments were artificially inoculated. The exact numbers of genotypes according to each category are included within brackets []. In boxplots, boxes enclose 50% of the central data, including median (black bold line) and mean (black diamond), while whiskers are ± 1.5 × interquartile range and dots represent extreme values. In the scatter plot, ** denotes the significance [-log_10_(p-value) = 128.4] of the correlation between YR scores from precision and large-scale screening experiments.

After outlier correction, heritability estimates for replicated experiments were in general higher than 0.7, with the only exception being SST_2018_5 (*h*^2^ = 0.54) (Table 4). The highest heritabilities (*h*^2^ = 0.92) were estimated in inoculated precision experiments ROS_ and WTZ_YR_2019. Due to material mislabeling during the prosecution of experiments, the cultivar ‘PilgrimPZO’ was completely discarded from further phenotypic analyses. The effective number of entries with YR scores (either BLUEs or single point values) ranged from 1,395 to 1,669 per individual screening experiment and between 722 and 797 for precision experiments. Because of the unbalanced structure of large-scale screening experiments, the pairwise entry overlap ranged between four and 1,641 common genotypes. In contrast, these numbers were higher in balanced precision experiments, ranging from 697 to 797 common entries between experiments. In our companion study^14^, PGR tested in the first five large-scale screening experiments constituted the base population for trait-customized core selection of the 600 SSD-PGR tested in precision experiments. For this reason, the pairwise entry overlap between large-scale screenings and precision experiments drops drastically from 259–339 to 13–26 in later experiments. All significant pairwise correlations (p-value < 0.05) between design-corrected YR scores from different experiments were positive and ranged between 0.29 and 0.92. In analyses across experiments, the heritability of YR-scores was 0.82 for the large-scale screening and 0.89 for precision experiments. Presumably due to unbalanced phenotyping, a very small proportion (0.4%) of BLUEs computed across large-scale screening experiments for 7,682 PGR plus 80 elite cultivars lied outside of the 1–9 parametric space (Fig. 4). This bias was not observed in the BLUEs computed across precision experiments for 199 elite cultivars and 600 SSD-PGR. Nevertheless, such a bias is ignorable considering the strong correlation [*r* = 0.77, -log_10_(p-value) = 128.4] of BLUEs across experiments for the overlapping material between precision and large-scale screening experiments. Large-scale screening experiments revealed that only a small PGR fraction (8.4%) have fewer infection symptoms than an average elite cultivar. We showed in our companion study^14^ that this resistant PGR fraction is enriched with material from European origins that entered the IPK genebank during recent decades. The implemented trait-customized core selection approach^14^ allowed to more than triple (27.5%) the PGR YR-resistant proportion in precision experiments while increasing exotic molecular diversity and reducing the association between population structure and trait variation. This provided the base to identify genetically diverse PGR donors of YR resistance sources not yet used in elite breeding^14^, which are being currently validated using classical and functional genetics approaches. Heritabilities, BLUEs and custom R codes for their computation are also available in the respective ‘BLUEs_and_heritabilities’ folders^71,72^.

**Table 4:** Heritabilities (*h*^2^) and matrix with the effective number of entries (underlined diagonal values) for outlier-and-experiment-design-corrected yellow rust (*Puccinia striiformis* f. sp. tritici) infections scored in 12 experiments of a large-scale screening and seven precision experiments as well as the significant correlations (p-value < 0.05, above diagonal) and number of overlapping entries (below diagonal) among them.

	Experiment^a^	Type^b^		Large-scale screening												Precision experiments														
			Experiment	GAT_2014_1	SST_2014_1	GAT_2014_2	GAT_2015_2	SST_2015_2	GAT_2015_3	GAT_2016_3	GAT_2017_3	GAT_2017_5	SST_2018_5	GAT_2019_6	SST_2019_6	GAT_YR_2018			ROS_YR_2018	WTZ_YR_2018			GAT_YR_2019			QLB_YR_2019			ROS_YR_2019	WTZ_YR_2019
			Type	1x	1x	1x	1x	1x	1x	1x	1x	1x	1x	M:2x	1x	E	L	M:2x	1x	E	L	M:2x	E	L	M:2x	E	L	M:2x	1x	1x
			*h*^2^																											
**Large-scale screening**	**GAT_2014_1**	1x	0.86	1514	0.76	0.85	0.79	0.71	0.87	0.51	—	—	—	—	—	0.65	0.63	0.67	0.74	0.68	0.64	0.64	0.53	0.55	0.54	0.57	0.54	0.58	0.69	0.70
	**SST_2014_1**	1x	0.84	1491	1514	0.79	0.54	0.60	0.73	0.53	0.64	—	—	—	—	0.62	0.61	0.65	0.62	0.72	0.68	0.68	0.50	0.54	0.54	0.51	0.50	0.55	0.72	0.69
	**GAT_2014_2**	1x	0.88	14	13	1544	0.60	0.70	0.84	0.78	0.65	—	—	—	—	0.55	0.49	0.51	0.59	0.59	0.60	0.60	0.51	0.49	0.50	0.34	0.42	0.44	0.64	0.63
	**GAT_2015_2**	1x	0.79	15	14	1502	1516	0.69	0.67	0.92	0.72	—	—	—	—	0.54	0.47	0.48	0.51	0.47	0.54	0.54	0.48	0.49	0.50	0.29	0.40	0.40	0.53	0.53
	**SST_2015_2**	1x	0.83	15	14	1453	1444	1467	0.76	0.51	—	—	—	—	—	0.50	0.46	0.47	0.50	0.64	0.57	0.57	0.42	0.44	0.44	0.34	0.38	0.40	0.62	0.64
	**GAT_2015_3**	1x	0.90	14	14	12	13	13	1579	0.76	0.75	—	—	—	0.55	—	0.52	0.52	—	—	—	—	—	0.54	0.56	0.61	—	0.59	—	0.54
	**GAT_2016_3**	1x	0.90	17	16	18	19	19	1525	1560	—	0.39	—	0.55	0.68	0.47	—	—	—	—	—	—	—	—	—	—	—	—	—	—
	**GAT_2017_3**	1x	0.86	16	15	18	19	19	1504	1512	1539	0.58	—	—	0.49	—	—	—	0.40	—	—	—	—	—	—	0.50	0.55	0.52	0.40	0.60
	**GAT_2017_5**	1x	0.76	7	7	5	6	6	24	26	24	1395	0.34	—	—	—	—	—	—	—	—	—	—	—	—	—	—	—	0.50	—
	**SST_2018_5**	1x	0.54	7	7	5	6	6	23	25	23	1363	1415	—	—	—	—	—	—	—	—	—	—	—	—	—	—	—	—	—
	**GAT_2019_6**	M:2x	0.74	8	8	4	5	5	20	22	20	23	23	1667	0.55	—	—	—	—	—	—	—	—	—	—	—	—	—	—	—
	**SST_2019_6**	1x	—	8	8	4	5	5	21	23	21	24	24	1641	1669	—	—	—	—	—	—	—	—	—	—	—	—	—	—	—
**Precision experiments**	**GAT_YR_2018**	E	0.91	282	282	330	331	319	19	26	26	16	15	14	14	779	0.80	0.88	0.67	0.50	0.59	0.59	0.70	0.76	0.76	0.52	0.53	0.55	0.61	0.61
		L	0.81	286	286	331	332	320	19	26	26	16	15	14	14	773	784	0.97	0.65	0.48	0.56	0.56	0.62	0.69	0.69	0.48	0.48	0.50	0.59	0.60
		M:2x	—	280	280	327	328	316	19	26	26	16	15	14	14	773	773	773	0.67	0.51	0.58	0.58	0.66	0.73	0.73	0.52	0.53	0.55	0.61	0.63
	**ROS_YR_2018**	1x	0.91	259	259	302	303	292	19	26	26	16	15	14	14	704	709	698	722	0.61	0.63	0.63	0.48	0.53	0.52	0.53	0.56	0.58	0.71	0.70
	**WTZ_YR_2018**	E	0.89	287	287	334	335	323	19	26	26	16	15	14	14	771	776	765	715	789	0.69	0.69	0.40	0.45	0.45	0.48	0.51	0.54	0.72	0.77
		L	0.88	285	286	328	329	317	19	25	25	16	15	14	14	756	761	750	703	768	775	1.00	0.48	0.54	0.54	0.44	0.47	0.50	0.72	0.69
		M:2x	—	282	283	324	325	313	19	25	25	16	15	14	14	750	755	744	697	768	768	768	0.48	0.54	0.54	0.44	0.47	0.50	0.72	0.69
	**GAT_YR_2019**	E	0.77	286	284	334	335	323	18	25	25	14	13	13	13	764	769	758	709	774	759	753	782	0.73	0.74	0.44	0.49	0.51	0.51	0.48
		L	0.85	285	285	332	333	321	19	26	26	15	14	13	13	766	771	760	708	776	761	755	771	784	1.00	0.47	0.52	0.53	0.54	0.56
		M:2x	—	281	279	328	329	317	18	25	25	14	13	13	13	753	758	747	698	763	748	742	771	771	771	0.47	0.51	0.52	0.53	0.55
	**QLB_YR_2019**	E	—	289	289	338	339	327	19	26	26	16	15	14	14	777	782	771	720	788	773	767	780	782	769	797	0.70	0.79	0.55	0.54
		L	—	289	289	338	339	327	19	26	26	16	15	14	14	777	782	771	720	788	773	767	780	782	769	797	797	0.97	0.58	0.57
		M:2x	—	289	289	338	339	327	19	26	26	16	15	14	14	777	782	771	720	788	773	767	780	782	769	797	797	797	0.62	0.61
	**ROS_YR_2019**	1x	0.92	283	283	335	336	324	19	26	26	16	15	14	14	766	771	760	713	777	764	757	769	771	758	784	784	784	787	0.84
	**WTZ_YR_2019**	1x	0.92	281	281	332	333	322	19	26	26	16	15	14	14	760	765	754	706	771	757	750	763	765	752	778	778	778	772	780

^a^With the exception of SST_2019_6 and QLB_YR_2019, where block-wise corrected data were respectively used, all comparisons were performed using best linear unbiased estimations.

^b^In experiments, either a single measurement (1x), both early (E) and late (L) measurements and/or the maximum value among them (M:2x) were used in comparisons.

### Yield breeding values of plant genetic resources to inform breeders and initiate pre-breeding programs

**Fig. 5:**
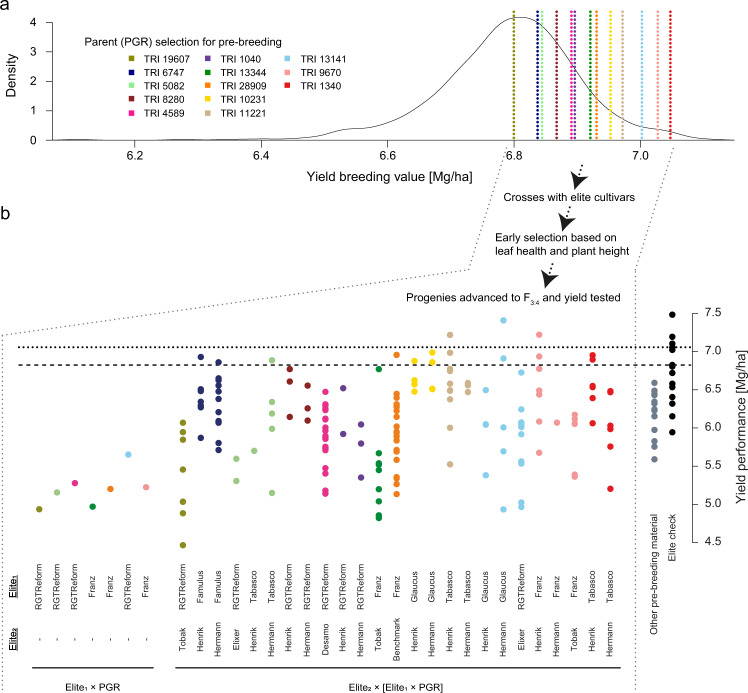
Using yield breeding value estimates of plant genetic resources (PGR) to initiate pre-breeding programs in wheat. (**a**) Kernel density distribution of yield breeding values (Mg/ha) for 707 PGR. Breeding values were estimated using yield data of ‘Elite × PGR’ F_1_ hybrids from 22 field experiments conducted between harvest years 2016 and 2020. Based on preliminary data from 2016, 13 PGR with superior breeding values were used as male parents in two- (Elite_1_ × PGR) and three-way (Elite_2_ × [Elite_1_ × PGR]) crosses involving 11 adapted elite cultivars. Vertical dashed lines indicate the breeding values of selected PGR estimated across the full set of 22 experiments. (**b**) After two-stage selection for high leaf health and reduced plant height, 173 advanced F_3:4_ PGR-derived progenies tracing back to 32 initial crosses were tested together with 15 elite cultivar checks (black dots) and 16 additional IPK pre-breeding lines (gray dots) in yield validation experiments conducted in two locations during harvest years 2020 and 2021. The best linear unbiased estimations of yield (Mg/ha) computed across validation experiments for the tested material are portrayed and grouped according to each initial cross. The color legend of PGR-derived populations matches that of the selected PGR parents used in initial crosses. Horizontal dotted and dashed lines indicate the yield performances of the best newest cultivar (‘LGCharacter’) and the mostly grown cultivar during the last decade (‘RGTReform’) in Germany, respectively.

After outlier-and-design correction, the effective number of entries with yield records ranged between 238 and 500 for individual breeding value estimation experiments. Due to the series-wise strategy to test plant material, the highest numbers of overlapping entries with yield records were observed between experiments conducted within the same year (Table 5). In more detail, the number of pairwise overlapping entries among 22 breeding value estimation experiments ranged between 0 and 500. All significant pairwise correlations (p-value < 0.05) between yield records of different experiments had positive sign, with magnitudes ranging between 0.11 and 0.96. Across experiments, 37 elite cultivars in addition to 227 PGR plus 1,429 ‘Elite × PGR’ and four ‘Elite_1_ × Elite_2_’ F_1_ hybrids have yield records in the outlier-and-design corrected dataset. Heritabilities of *per se* yield performance amounted to 0.89 for elite cultivars and PGR together, while the heritability of hybrid performance was 0.50, as also reported in our companion work^14^. The 1,429 ‘Elite × PGR’ originate from crossing 36 elite cultivars with 205 PGR and 510 SSD-PGR which, put together, trace back to 707 PGR tested in 1,427 merged ‘Elite × PGR’ hybrid crosses. Yield breeding values of PGR computed across the 22 estimation experiments ranged between 6.11 and 7.11 Mg/ha, with a mean of 6.79 (Fig. 5a). As reported in our companion work^14^, the heritability of breeding values was 0.32, which reflects the complexity of handling less than half of the genetic variation underlying yield in hybrids. A custom R code for breeding value estimation of PGR and heritability computations as well as its expected outputs are available in the ‘Breeding_values_and_heritabilities’ folder^73^.

**Table 5:** Matrix with the effective number of entries (underlined diagonal values) in, as well as significant correlations (p-value < 0.05, above diagonal) and number of overlapping entries (below diagonal) among 22 outlier-and-design-corrected experiments used to estimate the yield breeding value of 707 PGR using ‘Elite × PGR’ F_1_ hybrid crosses.

Experiment	BOH _2016	GAT _2016	HOH _2016	RNG _2016	SST _2016	ASD _2017	GAT _2017	HOH _2017	RNG _2017	SST _2017	GAT _2018	HDM _2018	HOH _2018	RNG _2018	SST _2018	GAT _2019	HDM _2019	HOH _2019	RNG _2019	SST _2019	GAT _2020	SST _2020
**BOH_2016**	240	0.91	0.85	0.83	0.90	0.35	0.47	0.32	0.26	0.26	—	—	0.72	0.93	—	—	—	—	—	—	—	—
**GAT_2016**	240	240	0.82	0.84	0.89	0.36	0.65	0.29	—	—	0.72	—	0.72	0.96	0.81	—	—	—	—	—	—	—
**HOH_2016**	240	240	240	0.79	0.83	0.29	0.59	0.39	0.28	0.27	0.67	—	—	0.92	—	—	—	—	—	—	—	—
**RNG_2016**	238	238	238	238	0.77	—	—	—	—	0.29	0.72	—	—	0.95	0.78	—	—	—	—	—	—	—
**SST_2016**	239	239	239	237	239	0.46	0.68	0.35	—	—	—	—	0.89	0.89	—	—	—	—	—	—	—	—
**ASD_2017**	94	94	94	94	94	353	0.62	0.58	0.65	0.30	—	—	—	—	—	—	—	—	—	—	—	—
**GAT_2017**	95	95	95	95	95	353	354	0.57	0.69	0.45	—	—	—	0.89	—	—	—	—	—	—	—	—
**HOH_2017**	93	93	93	93	93	338	339	340	0.58	0.17	—	—	0.79	—	—	—	—	—	—	—	—	—
**RNG_2017**	94	94	94	94	94	348	349	334	349	0.40	—	—	—	—	—	—	—	—	—	—	—	—
**SST_2017**	95	95	95	95	95	347	348	334	343	348	—	—	—	—	0.81	—	—	—	—	—	—	—
**GAT_2018**	9	9	9	9	9	8	8	7	7	8	292	—	0.54	0.56	0.49	—	—	—	—	—	—	—
**HDM_2018**	9	9	9	9	9	7	7	6	6	7	279	316	0.18	0.11	0.17	—	—	—	—	—	—	—
**HOH_2018**	9	9	9	9	9	8	8	7	7	8	289	313	328	0.80	0.53	—	—	—	—	—	—	—
**RNG_2018**	9	9	9	9	9	8	8	7	7	8	288	312	324	328	0.49	—	—	—	—	—	—	—
**SST_2018**	7	7	7	7	7	7	7	6	6	7	256	244	253	252	257	—	—	—	—	—	—	—
**GAT_2019**	0	0	0	0	0	0	0	0	0	0	0	0	0	0	0	374	0.32	—	0.51	0.53	—	—
**HDM_2019**	0	0	0	0	0	0	0	0	0	0	0	0	0	0	0	374	377	—	—	0.54	—	—
**HOH_2019**	0	0	0	0	0	0	0	0	0	0	0	0	0	0	0	320	322	322	—	0.17	—	—
**RNG_2019**	0	0	0	0	0	0	0	0	0	0	0	0	0	0	0	363	366	312	366	0.29	—	—
**SST_2019**	0	0	0	0	0	0	0	0	0	0	0	0	0	0	0	372	375	320	364	375	—	—
**GAT_2020**	0	0	0	0	0	0	0	0	0	0	0	0	0	0	0	0	0	0	0	0	500	0.55
**SST_2020**	0	0	0	0	0	0	0	0	0	0	0	0	0	0	0	0	0	0	0	0	500	500

Based on preliminary results from estimation experiments conducted during harvest year 2016, 13 PGR were selected for their superior breeding value to initiate a small pre-breeding program using 11 European elite cultivars as breeding value receptors (Fig. 5). Computed across the 22 estimation experiments, breeding values of selected PGR ranged from 6.80 to 7.05 Mg/ha and were superior to the general mean of breeding value estimates (Fig. 5a). Progenies from seven and 25 Elite_1_ × PGR and Elite_2_ × [Elite_1_ × PGR] initially performed crosses, respectively, were advanced to F_3:4_ and pre-selected for good visual performance. The in total 173 advanced F_3:4_ progenies, with at least one progeny per initial cross, were tested for grain yield together with 15 elite cultivar checks and 16 additional pre-breeding lines in four validation experiments conducted in two locations during harvest years 2020 and 2021 (Fig. 5b). A custom R code for BLUEs and heritability computation as well as its expected output files are available in the ‘BLUEs_and_heritabilities’ folder^74^. Yield performances were highly repeatable, which was reflected by the heritabilities within (*h*^2^ ≥ 0.76, Table 6) and across (*h*^2^ = 0.76) validation experiments as well as by the significant positive correlations among them (*r* ≥ 0.68, Table 6). The highest yields were observed in general for elite check cultivars, with grain yield values ranging between 5.94 and 7.48 Mg/ha (Fig. 5b). For them, the year of cultivar release was significantly and positively correlated with the yield performance (*r* = 0.79, p-value < 0.001), reflecting the advances in yield breeding achieved between years 2007 and 2020. Among the 173 + 16 = 189 advanced F_3:4_ progenies, up to two with an average of only 1.1 progenies per initial Elite_1_ × PGR cross made it through the first selection stages based on visual performance. Adding a second elite cultivar to pedigrees as Elite_2_ × [Elite_1_ × PGR] increased in general the number of progenies per initial cross with good visual performance to an average of 6.7, with the least and most prolific crosses having one and 21 F_3:4_ progenies, respectively. Two-way crosses were as a group also significantly less competitive than three-way crosses (${\widehat{\mu }}_{Three-way}-{\widehat{\mu }}_{Two-way}$ = 0.72 Mg/ha, p-value < 0.001). The lower outputs for two-way crosses could be attributed to the increased proportion of deleterious PGR background still present in them, which also indicates that a realistic use of PGR variation for pre-breeding is achieved through three-way crosses. In fact, three-way crossing schemes are already the main strategy to introduce PGR variation into large-scale global pre-breeding programs such as Seeds of Discovery^22^ (https://seedsofdiscovery.org/). Although none of the PGR-derived progenies was as competitive as the best check cultivar ‘Informer’ (released in 2018), among the 173 F_3:4_ progenies whose PGR parents were selected based on breeding values, three (2%) and 15 (9%) had better yield performance than the best newest (‘LGCharacter’, released in 2020) and the locally most grown cultivar (‘RGTReform’, released in 2014), respectively. Regarding the additional 16 pre-breeding lines whose PGR parents lack of breeding value estimates, none of them could reach these previously mentioned yield levels. Global efforts of the Seeds of Discovery initiative led to the development of 2,867 pre-breeding lines that trace back to 366 exotic wheat PGR^22^. Multiple environment yield trials conducted in Central America and South Asia revealed that locally, up to ~2% of these pre-breeding lines have better yield performance than the best adapted varieties. Moreover, pre-breeding lines with beneficial traits trace back to 62 of the 366 exotic founders (17%) used as PGR parents by Seeds of Discovery^22^. In our small pre-breeding program established using breeding values as parent selection tool for PGR (Fig. 5**)**, competitive pre-breeding lines trace back to eight of the 13 selected PGR (62%) parents. All in all, at least as regards visual performance and yield, our validation experiments suggest that using breeding values as tool could boost the input-to-output ratio for pre-breeding programs.

**Table 6:** Heritabilities (*h*^2^) and matrix containing the effective numbers of entries (underlined diagonal values) for best linear unbiased estimates of yield in four pre-breeding validation experiments as well as the significant correlations (p-value < 0.05, above diagonal) and number of overlapping entries (below diagonal) among them.

Experiment	*h*^2^	Experiment			
		GAT_PB_2019	SST_PB_2019	GAT_PB_2020	SST_PB_2020
**GAT_PB_2019**	0.91	95	0.72	—	—
**SST_PB_2019**	0.85	56	57	—	—
**GAT_PB_2020**	0.87	3	3	118	0.68
**SST_PB_2020**	0.76	3	3	108	108

### High genomic prediction accuracies support the suitability of data for integrated phenotypic-genomic analyses

**Fig. 6:**
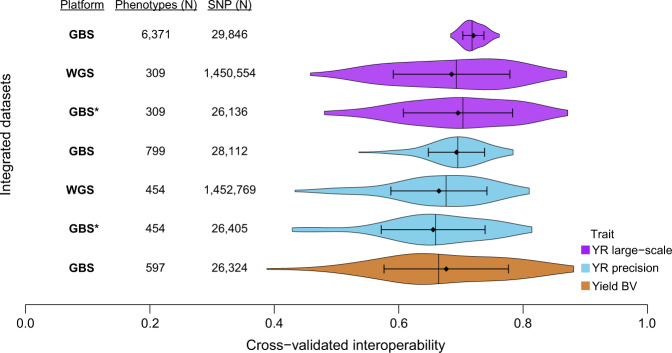
Distributions of cross-validated interoperability between genomic and phenotypic data. Genotyping platforms were genotyping-by-sequencing (GBS) and whole-genome sequencing (WGS, 3-fold coverage), while phenotypes corresponded to the best linear unbiased estimates for yellow rust (YR) scorings computed across large-scale screening or precision experiments, as well as yield breeding values (BV) computed across estimation experiments. Interoperability was estimated as the genomic prediction accuracy using 80% of the integrated data as training and 20% as validation set in 100 cross-validation runs. Total number (N) of samples with phenotypes and polymorphic SNP markers used for cross-validations according to each genotyping platform are portrayed as table on the left side. In case of GBS*, the same training and validation phenotypes used for WGS were considered. For more details on cross-validations, please see Methods. In distributions, diamonds, horizontal and vertical lines correspond to the average, standard deviation and median, respectively. Violin plots were obtained using the vioplot R package (v0.3.7).

Seed mixtures, sample mislabeling, among other sources of systematic errors can occur in large-scale characterizations. This obviously disrupts the connectivity between genotype and phenotype and in turn, decrease the value of the data for integrated analyses. To rule-out the presence of such data-imparity, we used the cross-validated accuracy of genomic prediction as a quality measure for genomic-phenotypic data interoperability (Fig. 6).

Integrating YR-scorings and GBS data resulted in 6,371 and 799 genotyped samples having records in large-scale screenings and precision experiments, respectively (Fig. 6). In cross-validations, the genomic-phenotypic data interoperability between YR-scorings and GBS was in general high, with prediction accuracies of 0.72 ± 0.02 and 0.69 ± 0.05 for large-scale screening and precision experiments, respectively. As expected from past simulation studies^80,81^, the slightly higher accuracy for large-scale screenings than for precision experiments can be attributed to the ~8 times larger population size of the former group, which provided in turn also a small increase (~6%) in the number of polymorphic markers used for prediction. Shifting to WGS decreased population sizes for large-scale screenings and precision experiments to 309 and 454 samples but accuracies only slightly dropped to 0.69 ± 0.09 and 0.66 ± 0.08, respectively. Moreover, the ~55-fold increase in number of polymorphic markers from WGS provided practically no improvement in accuracy over GBS when the same population size was considered for both genotyping platforms (see GBS* in Fig. 6). This last observation is not surprising considering that GBLUP^60^ mostly relies on relatedness for prediction^80,81^ and that, as mentioned before, GBS- and WGS-based Rogers’ distances were highly correlated. Although out of the scope of this work, we anticipate that alternative genomic prediction methods less dependent on relatedness^80,81^ would benefit more from the increased marker densities provided by WGS.

GBS samples of PGR having also breeding value estimates amount to 597 (Fig. 6). Prediction accuracies of 0.68 ± 0.1 support also the high genomic-phenotypic data interoperability for these samples and come very close to the estimates presented in our companion study using 1,000 instead of 100 cross-validations^14^. Last but not least, only 24 PGR samples have both WGS data and breeding value estimates – a too limited number of genotypes to meaningfully assess the genomic-phenotypic data interoperability. Although integrated analyses for WGS and yield breeding values are currently not advisable, PGR are available upon request, and we thus fully encourage future activities that increase the connectivity between these two types of data. A custom R code to assess the genomic-phenotypic data interoperability as well as its needed inputs and expected outputs are available here^70^.

## Usage Notes

We expect that these FAIR data support and encourage future research and breeding initiatives that further valorize crop plant genebanks. The genebank material of the ‘TRI’ catalogue is available upon request using IPK genebank DOIs and can be accessed through GBIS (gbis.ipk-gatersleben.de) under the conditions of a standard material transfer agreement (SMTA). Seeds of field isolated accessions and pre-breeding material can be requested upon availability through their SSD- and FAMILY-DOIs, respectively, by following SMTA conditions as well (contact e-mail: reif@ipk-gatersleben.de).

## Data Availability

The custom awk script for filtering of VCF files is available at e!DAL-PGP and can be accessed here^82^. Custom R codes for phenotypic parameter estimations are included within the respective ‘R_code’ subfolder of each dataset^71–74^ deposited into e!DAL-PGP. In addition, custom R codes to assess the genomic-phenotypic data interoperability and the computation of Roger’s distances as well as their needed inputs and expected outputs were also deposited into e!DAL-PGP and can be accessed here^70^.
